# Effects of Counselling on Quality of Life among Cancer Patients in Malaysia: A Randomized Controlled Trial

**DOI:** 10.18502/ijph.v49i10.4693

**Published:** 2020-10

**Authors:** Ummavathy PERIASAMY, Sherina MOHD-SIDIK, Mehrnoosh AKHTARI-ZAVARE, Lekhraj RAMPAL, Siti Irma Fadhilah ISMAIL, Rozi MAHMUD

**Affiliations:** 1. Hospital Tuanku Jaafar, Seremban, Negeri Symbian, Malaysia; 2. Cancer Resource & Education Center, Universiti Putra Malaysia, 43400 Serdang, Selangor, Malaysia; 3. Department of Psychiatry, Faculty of Medicine & Health Sciences, Universiti Putra Malaysia, 43400 Serdang, Selangor, Malaysia; 4. Department of Public Health, Faculty of Health, Tehran Medical Sciences, Islamic Azad University, Tehran, Iran; 5. Department of Community Health, Faculty of Medicine & Health Sciences, Universiti Putra Malaysia, 43400 Serdang, Selangor, Malaysia

**Keywords:** Quality of life, Cancer patients, Pharmacist, Counselling, Malaysia

## Abstract

**Background:**

We aimed to assess whether “Managing Patients on Chemotherapy” book is effective to improve quality of life (QOL) of cancer patient via counselling by pharmacist.

**Methods:**

A randomized control trial study was run among 2120 cancer patients in public hospitals in Peninsular Malaysia, from Apr 2016–Jan 2018. The treatment group received counselling regarding chemotherapy by using developed module. The data were collected at three time-points: baseline, 1^st^, 2^nd^ and 3^rd^ follow-ups after counseling by Validated Malay version of the WHOQOL-BREF of questionnaire. Data analyses were done using χ^2^ and two-way repeated measure ANOVA.

**Results:**

The treatment group improved significantly as compared to control group in physical health, psychological health, social relationship, environment and overall QOL (*P*<0.00).

**Conclusion:**

The “Managing Patients on Chemotherapy” book along with repetitive counselling by pharmacists is a useful intervention for improving QOL of cancer patients undergoing treatment.

## Introduction

Globally, one of the reason of death and morbidity is cancer, with approximately cause 8.8 million deaths in 2015 (1, 2). Based on the WHO, 70% of cancer death occur in low and middle income countries (2). In the period of 2007–2011, 103,507 new cases of cancer and 64,275 cancer mortality were reported in Malaysia, which had increased fivefold from 2003 (3, 4).

Most common types of cancer treatment (chemotherapy and radiotherapy) which increase patients’ survival rates but is associated with serious side effects and negative effect on their QOL (5). In the treatment of cancer patients, it is important not only to deduct the death rate, prevent recurrence of cancer, and other complications which experience by caregivers, but also improve QOL of cancer patients (6, 7).

QOL is defined as the patient’s perception of self-wellbeing and contains several aspects of functioning including psychological, physical, cognitive and social functioning (8). Newly, QOL has been consider as a main goal for measuring the level of the care and management in oncology medicine (9), also found to be an important predictor of survival in numerous studies worldwide which include Scotland (10), Malaysia (11) and China (12). Therefore, QOL among cancer patients must considered by physicians before commencement of treatment of cancer patient.

Chemotherapy counselling for patients before start their treatment is crucial and has a positive impact on the cancer patient’s QOL (5). Through counselling patients improve their knowledge about the process of treatment, side effects, along with reduction of distress surrounding chemotherapy treatment (5). Nowadays, in most countries the role of pharmacists are changing from traditional drug services towards patient-oriented services such as providing information about chemotherapy regimens and potential side effects for cancer patients (13, 14). In 2014, a book titled “Managing Patients on Chemotherapy” (MPCH) was published in Malaysia (15). This book focused on counselling of patients which undergoing chemotherapy treatment via pharmacists; it was the first book of its kind ever to be published in Malaysia (15).

Based on the findings of a preliminary study (16) we designed a randomized controlled trial to implement and assess the effectiveness of chemotherapy counselling by pharmacists on QOL of cancer patients based on the MPCH book in selected public hospitals in Peninsular Malaysia.

## Materials and Methods

### Study design and participants

A randomized control trial study (RCT) with The ANZCTR clinical trial registry (ACTRN12618001345279), was conducted to measure the effectiveness of chemotherapy counselling by pharmacist among cancer patients from Apr 2016 through Jan 2017. All Malaysian cancer patients (all types), in stage I, II of cancer, aged 18 yr and over and those on the 1^st^ and 2^nd^ rounds of chemotherapy treatment in public hospitals with oncology facilities in Peninsular Malaysia were recruited. Patient with communication problem, psychiatric disorders and those under third round of chemotherapy onwards were not eligible to participate.

### Sampling method and Randomization

The multistage random sampling method was conducted for selecting participants. Firstly, ten out of thirteen states randomly were chosen in Peninsular Malaysia. Then, ten government hospitals (one hospital for each state) was chosen by simple random sampling from list of public hospitals which was achieved from the Ministry of Health (MOH), Malaysia. The list of patients who met the inclusion criteria was obtained from the cytotoxic drug reconstitution, Pharmacy Department of each selected hospital and served as a sampling frame. The site investigators assigned participants to treatment or control group by using odd or even number, respectively. Patient recruited based on the number of registered patients in each hospital and on a daily basis. Respondents were chosen from different types of hospital wards for controlling contamination.

### Educational development

One pharmacist (the person who doing consultation) were consulted patients based on the “MPCH” book. The content of this book included sections from an earlier education module on chemotherapy counselling which mention in Table 1.

**Table 1:** Content of “Managing Patients on Chemotherapy” Educational Module

***Chapter***	***Content***
Preface	
Chapter 1 introduction	About chemotherapy Causes of chemotherapy side effects Chemotherapy drugs and potential side effects
Chapter 2 Get ready for chemotherapy	Before, during and after chemotherapy
Chapter 3 handling chemotherapy side effects	Nausea and vomiting Hair loss/ Fatigue/anemia Infection / Bleeding/ constipation Mouth, gums, throat problems appetite changes/ weight gain/ pain
Chapter 4 Chemotherapy and psychological issue	Depression Anxiety and fear Managing adverse psychological effects of cancer anger Managing complications due to cytotoxic extravasation

A complete description of the development of this book has been published elsewhere (16, 17). This book was developed and written by the specialists in the fields of pharmacist, oncologist, dietitian, clinical psychologist and public health physician. Regarding developing this book a focus group discussion (FGD) was conducted among a group of cancer patients (16), adding some information from module of the National Cancer Institute (NCI) entitle “Chemotherapy and You” (18) and then added comments which obtained from pharmacist who had experience of working in different chemotherapy wards. Finally, the final version of the book was pre-tested among forty cancer patients which not included in actual study. This eventually led to the publication of the book titled “Managing Patients on Chemotherapy” by Periasamy et al (15).

### Intervention-patient counselling

Treatment group received all parts of the book through counselling which conducted by one pharmacist who is qualified to run these sessions. Chemotherapy consultation sessions which was done in three consecutive chemotherapy rounds and each session was around one hour.

Patients asked their questions at the end of session. Patients in the control group received the usual care, which include basic information for coping any side effects of chemotherapy only in the first session of their treatment by pharmacists. Control group received the “MPCH” book at the end of study. For controlling participant retention in this study a gift was given to each participant after they had completed the study. The detail information of intervention has been published elsewhere (16,17).

### Assessment

Assessments outcome was done prior to chemotherapy (baseline, T0) and after first round of chemotherapy (T1) (± 3–6 weeks), second round of chemotherapy (T2) (±3–6 weeks) and third round of chemotherapy (T3) (±3–6 weeks). Due to the different times of treatment for each participant, the duration of data collection for each cancer patient varied between 12–18 weeks (Table 2).

**Table 2:** Study schedule and measurements used

***Time point***	***Study period***						

	***Enrolment -T_1_***	***Allocation T_A_***	***Baseline T_0_***	***Intervention***	***Follow up***		

					***T_1_***	***T_2_***	***T_3_***
Patient Enrolment							
Eligibility screening	×						
Informed consent	×						
Allocation		×					
Intervention							
Counselling				×			
Instrument							
Socio-demographic			×				
WHOQOL-BREF			×		×	×	×

-T_1_, during inpatient treatment; T_0_, during inpatient treatment; T_A_, allocation to intervention or control group; T_A_, Intervention; T_1_, first follow up; T_2_, second follow up, T_3_, third follow up

### Outcome measure

QOL of cancer patients which doing chemotherapy treatment were outcomes of this study. Data were collected via a validated self-administered questionnaire which translated to local language (Malay) (19).

In the follow-ups (1^st^, 2^nd^ and 3^rd^), patients were exposed to the same preliminary questions in the baseline questionnaire, except for questions related to socio-demographic characteristics, which were only collected at baseline. Questionnaire were completed by patients in their respective wards before starting their chemotherapy treatment on that day.

### Socio-demographic information

Items on socio-demographic included age, gender, ethnicity, religion, marital status, education, family income, number of children, and working status. Also, we included some clinical information such as; cancer stage, type of treatment, mentally disturbed, pain due to cancer.

### WHO Quality of Life-BREF (WHOQOL-BREF)

QOL was measured by the 26-items of WHOQOL-BREF which measuring four constructs of QOL such as; physical health, social relationship, psychological health and environment (20). Likert Scale (1) “very poor” to (5) “very good”, with rang score from 26 to 130 were used as response categories. All construct of QOL don’t have any cut-off points and measured in a positive way (i.e. higher numbers denoting greater QOL). For the current study, the validated Malay version of the WHOQOL-BREF with acceptable intra-class correlation coefficient (ICC) value from 0.79–0.88 was used (19).

### Sample size

The sample size was determined by considering group difference of 20% (24) by using the Rosner formula (21). In order to obtain 90% power (*P*=0.05) with considering of 20% attrition rate, 2140 cancer patients from all ten public hospitals were selected. Twenty patients refused to participate in this study because of change of place (hospital), no more interest, and not feeling well. Therefore, only 2120 cancer patients completed three times ongoing counselling (response rate 99.11%).

### Ethical approval

This study was approved by the MOH, Malaysia, Ethical Committee of Universiti Putra Malaysia (UPM), and each of the ten selected hospitals. All participants were informed orally about this study and the informed consent was taken from them before conducting the study.

### Statistical Analysis

Data were analysed using SPSS version 22(Chicago, IL, USA). Differences among two groups were ascertained by Chi-square. Regarding to evaluate the changes in the mean score of each construct of QOL between two groups from baseline until third follow ups, the two-way repeated measure ANOVA was used. To find out which group time the statistically differences actually occurred; Post hoc analysis was run based on a new *P*<0.005 after Bonferroni adjustment.

## Results

### Baseline data

A total of 2120 out of 2140 cancer patients participated, with 1060 respondents in each group. Most of the respondents were female (56.7%), Malay (63.2%) and married (68.9%). The detail information of characteristics of cancer patients are presented in Table 3. Baseline demographic and clinical factors were well balanced between the two groups and not significant was found between two groups at baseline (Table 3).

**Table 3:** Socio-demographic characteristics of respondents (n=2120)

***Characteristics***	***Intervention group***	***Control group***	***Total participants n(%)***	***Statistics***

	***n(%)***	***n(%)***		
Age (yr)				
<45	117(11.0)	148(14.0)	265(12.5)	χ2 =5.31, p=0.15
45–54	174(16.4)	185(17.5)	359(16.9)	
55–64	343(32.4)	332(31.3)	675(31.8)	
>65	426(40.2)	395(37.3)	821(38.7)	
Gender				
Male	443(41.8)	474(44.7)	917(43.3)	χ2 =1.84, p=0.17
Female	617(58.2)	586(55.3)	1203(56.7)	
Race				
Malay	680(64.2)	659(62.2)	1339(63.2)	χ2=0.89, p=0.34
Non-Malay	380(35.8)	401(37.8)	781(36.8)	
Religion				
Muslim	680(64.2)	380(35.8)	1338(63.1)	χ2=0.98, p=0.32
Non-Muslim	658(62.1)	402(37.9)	782(36.9)	
Marital Status				
Single	78(56.1)	61(43.9)	139(6.6)	χ2=3.91, p=0.14
Married	711(48.7)	749(51.3)	1460(68.9)	
Others	271(52.0)	250(48.0)	521(24.6)	
Education level				
Illiterate	225(46.3)	261(24.6)	486(22.9)	χ2 =5.84, p=0.06
Diploma & less	648(52.2)	594(56.0)	1242(58.6)	
Degree & above	392(18.5)	205(19.3)	392(18.5)	
Family Income (RM)				
No income	389(36.7)	389(36.7)	778(36.7)	χ2 =0.83, p=0.84
<1500 RM	191(18.0)	193(18.2)	384(18.1)	
1501–3500 RM	280(26.4)	265(25.0)	545(25.7)	
>3501 RM	200(18.9)	213(20.1)	413(19.5)	
Number of child				
No child	131(48.9)	137(51.1)	268(12.6)	χ2 =7.06, p=0.07
1–2 child	330(47.9)	359(52.1)	689(32.5)	
3–4 child	342(48.9)	357(51.1)	699(33.0)	
>5 child	257(55.4)	207(44.6)	464(21.9)	
Working status				
Yes	472(44.5)	445(42.0)	917(43.3)	χ2 =2.51, p=0.28
No	389(36.7)	389(36.7)	778(36.7)	
Retired	199(18.8)	226(21.3)	425(20.0)	
Type of cancer				
Breast	359(33.9)	323(30.5)	682(32.7)	χ2 =11.81, p=0.06
Cervix	101(9.5)	97(9.2)	198(9.3)	
Ovarian	36(3.4)	43(4.1)	36(3.4)	
Colorectal	298(28.1)	299(28.2)	298(28.1)	
Lymphoma	74(7.0)	58(5.5)	132(6.2)	
Stomach	83(7.8)	120(11.3)	203(9.6)	
Others	109(10.3)	120(11.3)	229(10.8)	
Cancer Stage				
Stage 1	100(9.4)	108(10.2)	208(9.8)	χ2 =2.50, p=0.47
Stage 2	165(15.6)	158(14.9)	323(15.2)	
Stage 3	407(38.4)	378(35.7)	785(37.0)	
Stage 4	388(36.6)	416(39.2)	804(37.9)	
Type of cancer treatment				
Chemotherapy	964(90.9)	964(90.9)	1944(91.7)	χ2 =1.58, p=0.20
Chemotherapy & radiation	96(9.1)	96(9.1)	176(8.3)	
Pain due to cancer				
Yes	555(52.4)	580(54.7)	1135(53.5)	χ2 =1.18, p=0.27
No	505(47.6)	480(45.3)	985(46.5)	
Mentally disturbed				
Yes	1060(100)	1060(100)	2120(100)	χ2 =N/A+, p=0.27
No	0(0)	0(0)	0(0)	

SD standard deviation;

* Significant at level *P*< 0.05; +Fisher’s exact test

### Change in QOL and each domain

Table 4 are presented the mean scores of QOL and each domain score in both groups at baseline until 3^rd^ follow up. At baseline, there were no statistically significant differences between overall mean score of QOL and each domain score between the both groups. However, the mean differences of overall QOL and each domain for the intervention group was significantly higher compared to the control group from baseline until 3^rd^ follow up.

**Table 4:** Change in QOL and each domain between intervention and control group at baseline until 3^rd^ follow ups

***Quality of Life***	***Baseline***	***1^th^ follow-up***	***2^nd^ follow up***	***3^rd^ follow up***	***Effect of intervention***	***Statistics***

	***Mean ± SD***				***Mean differences (95%CI)***	
Physical health						
Intervention Group	64.31 ± 21.25	69.36 ± 20.29	74.01 ± 16.85	82.68 ± 14.63	20.83, (19.84–21.82)	0.000^*^
Control group	64.68 ± 19.68	59.29 ±19.88	59.29 ± 16.71	38.35 ± 14.93	0	
Psychological health						
Intervention Group	59.84 ± 18.79	59.30 ± 19.51	65.48 ± 18.89	75.55 ± 14.49	16.77, (15.80–17.74)	0.000^*^
Control group	61.43 ± 19.34	54.77 ± 19.09	41.50 ± 17.09	35.36 ± 14.12	0	
Social relationships						
Intervention Group	59.71 ± 23.75	67.09 ± 21.80	72.32 ± 19.66	84.31 ± 9.05	24.58, (23.47–25.69)	0.000^*^
Control group	57.71± 23.94	54.68 ± 22.52	38.17 ± 17.17	34.55 ± 16.16	0	
Environment						
Intervention Group	63.60 ± 21.09	69.86 ± 20.67	74.44 ± 17.17	87.33 ± 10.23	21.99, (21.00–22.98)	0.000^*^
Control group	62.76± 20.07	59.71 ± 19.55	45.09 ± 16.61	39.69 ± 15.19	0	
Overall QOL						
Intervention Group	247.48±76.35	265.63±77.29	286.26±53.88	329.89±30.88	84.19, (80.62–87.75)	0.000^*^
Control group	246.59±73.18	228.46±72.65	169.48±57.74	147.97±37.82	0	

* Significant at level *P*< 0.05

The results of the two-way repeated measure ANOVA analysis for overall QOL and each domain on both groups and time (baseline until 3^rd^ follow up) effects and interaction between group and time showed that; in each domain of QOL and also overall QOL; there were significant main effect for group, time and interaction between group and time.

### Improved in Mean overall QOL and each domain scores within group counselling sessions (over time)

To find out where the actual differences occurred pairwise comparison of baseline until 3^rd^ follow up on overall QOL and each domain scores was conducted. The differences in satisfaction scores were considered significant at (*P*=0.005) after Bonferroni adjustment. All domains of QOL significantly improved after counselling sessions (*P*<0.000) except baseline to 1^st^ counselling session (*P*=1.00) and baseline to 3^rd^ counselling session (*P*=0.06) for physical health, baseline to 1^st^ counselling session (*P*=0.08) and baseline to 3^rd^ counselling session (*P*=1.00) for environment, and baseline to 1^st^ counselling session (*P*=1.00) for overall QOL which were not significant (Table 5).

**Table 5:** Pairwise Comparison of overall QOL and each domain scores at different levels trials

***(I) time***	***(J) time***	***Physical health***	***Psychological health***	***Social relationships***	***Environment***	***Overall QOL***
Baseline	1^st^ follow-up	*P*=1.00	*P*=0.00^*^	*P*=0.00^*^	*P*=0.08	*P*=1.00
	2^nd^ follow-up	*P*=0.00^*^	*P*=0.00^*^	*P*=0.00^*^	*P*=0.00^*^	*P*=0.00^*^
	3^rd^ follow-up	*P*=0.06	*P*=0.00^*^	*P*=0.00^*^	*P*=1.00	*P*=0.00^*^
1^st^ follow-up	Baseline	*P*=1.00	*P*=0.00^*^	*P*=0.00^*^	*P*=0.08	*P*=1.00
	2^nd^ follow-up	*P*=0.00^*^	*P*=0.00^*^	*P*=0.00^*^	*P*=0.00^*^	*P*=0.00^*^
	3^rd^ follow-up	*P*=0.00^*^	*P*=0.02^*^	*P*=0.00^*^	*P*=0.00^*^	*P*=0.00^*^
2^nd^ follow-up	Baseline	*P*=0.00^*^	*P*=0.00^*^	*P*=0.00^*^	*P*=0.00^*^	*P*=0.00^*^
	1^st^ follow-up	*P*=0.00^*^	*P*=0.00^*^	*P*=0.00^*^	*P*=0.00^*^	*P*=0.00^*^
	3^rd^ follow-up	*P*=0.00^*^	*P*=0.00^*^	*P*=0.00^*^	*P*=0.00^*^	*P*=0.00^*^
3^rd^ follow-up	Baseline	*P*=0.06^*^	*P*=0.00^*^	*P*=0.00^*^	*P*=1.00	*P*=0.00^*^
	1^st^ follow-up	*P*=0.00^*^	*P*=0.02^*^	*P*=0.00^*^	*P*=0.00	*P*=0.00^*^
	2^nd^ follow-up	*P*=0.00^*^	*P*=0.00^*^	*P*=0.00^*^	*P*=0.00^*^	*P*=0.00^*^

* Significant at level *P*< 0.005

### Changes in each domain of QOL in two groups counselling sessions (over times)

**Fig. 1:**
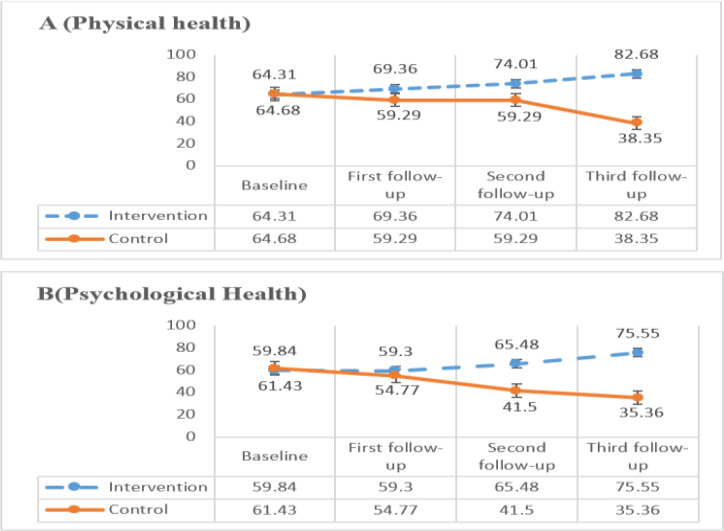
Comparison of changes in physical health and psychological health domains of QOL (A, B) in intervention and control group over times. Baseline: Before doing counselling, First: After doing first counselling session, Second: After doing second counselling session, Third: After doing third counselling session

**Fig. 2:**
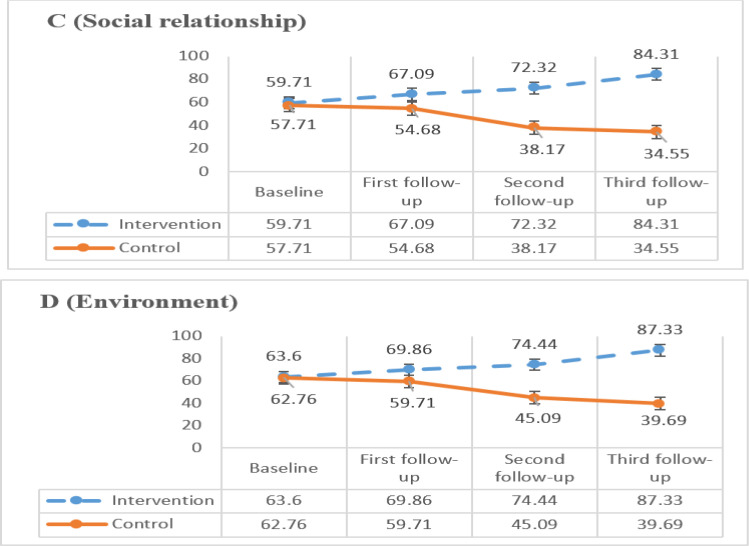
Comparison of changes in social relationship and environment domain of QOL (C, D) in intervention and control group over times. Baseline: Before doing counselling, First: After doing first counselling session, Second: After doing second counselling session, Third: After doing third counselling session

Mean changes from baseline until 3^rd^ follow-up in each domain of QOL for both groups are shown in Fig. 1 and 2. Based on the figures there was significant improvement in mean score of each domain of QOL from baseline until 3^rd^ follow-up in the treatment group after each counselling session(*P*=0.000). However, the mean score of each domain of QOL was decrease in control group after 3^rd^ follow up.

## Discussion

There was a significant betterment in QOL of cancer patients after ongoing counselling session, which can concluded QOL of cancer patients on the first and second round of treatment can be improved by providing ongoing chemotherapy counselling by pharmacist.

### Quality of life

Few studies highlighted that cancer affects patients’ QOL and their health (22, 23). Overall, this study found that the treatment group had higher mean score of each domain and overall QOL as compared to the control group (*P*<0.000); which highlighted the effectiveness of ongoing counselling by pharmacist based on the “‘MPCH book’”. This finding is similar with findings of study which done in Malaysia (24) and its result showed spending time with patients and ongoing counselling cause better understanding of patients from their disease which positively improve QOL of them.

### Psychological health and social relationship

The diagnosis and treatment of cancer can effect change in self-esteem and sexual function, by causing patients huge damage in psychology and social relationships (25, 26). Psychosocial intervention via education or counselling have a positive impact on psychology and social relationships of cancer patients (27, 17). Most importantly, and in accordance with other studies, our results indicate that psychological health and social relationships of cancer patients improved significantly over time after doing repetitive counselling by pharmacists which guided via MPCH book (16, 28). This is supported by the fact that in western countries, for improving QOL of cancer patients, health care providers can used many developed clinical practice guidelines which focus on psychotherapy and supportive care (29).

### Environment and physical health

As in our analysis, significant difference was found between groups over time in environment and physical health constructs of QOL (*P*<0.000). Similarly, participants in the counselling sessions lead to an improvement in the environment and physical health constructs of QOL (18). In line with this study, Jacobsen et al also highlighted physical health and physiological health of cancer patients could positively effect by providing counselling by physicians (30). Regarding environment and physical health constructs of QOL in this study, no significant improvement was not found in baseline to 1^st^ and 3^rd^ follow ups. With regards to physical health, this may be because from baseline to 1^st^ follow-up the duration is too short to see any changes; whereas from baseline to 3^rd^ follow-up, physical health of some of the patients may have deteriorated due to their cancer and increase their chemotherapy side effects. As for the environment construct of QOL, we feel that ongoing counselling with more follow-ups may show more improvement; however this improvement may occur well after four, five or even more counselling sessions and needs to be proven in other studies.

### Role of pharmacist

The role of a pharmacist does not only involve prescription of medicine for the treatment of cancer as traditionally perceived, but should also include appropriate counselling and information for cancer patients (31). Counselling can provide mental health support for them during treatment and improve their QOL (31). In this regard, pharmacists play an essential role in counselling of cancer patients, because they are uniquely trained to know all medications prescribed to a patient and how these interact with the cancer treatment regimen (32). Our results highlighted pharmacists playing important role in improving QOL of cancer patients during their treatment through ongoing counselling. In this line, results of studies done in Malaysia (17) and Spain (33) showed that providing counselling by pharmacist for cancer patients during their chemotherapy had affirmative impact on physical and mental health; consequentially improves QOL.

### Limitations and strengths of study

Using RCT as a gold standard, large sample size and very low attrition rate are the power of this study. On the other and, the use of a validated Malay version of questionnaire facilitated the findings of QOL among the cancer patients. Based on the literature presumably this is the first national study on chemotherapy counselling conducted among oncology patients in Malaysia. Consequently, the findings can be used as a fundamental for further research and plays an important role for policy makers, where a policy change can be established to implement counselling for all cancer patients while they undergo chemotherapy. This study also has some significant limitations; our study run among cancer patients so this results cannot be generalized to another type of disease. Consequently, further research among patients with another type of disease is needed to find out the role of pharmacist as a consultant person. Secondly, no objective measures to evaluate the respondents because all data were self-reported.

## Conclusion

Ongoing counselling by pharmacist improved QOL of cancer patients undergoing chemotherapy. The four dimensions of QOL improved with pharmacist counselling at the start of systemic therapy. Consequently, it is suggested that providing counselling sessions during cancer patients’ treatment by pharmacists with the aim of improving their QOL during and after treatment.

## Ethical considerations

Ethical issues (Including plagiarism, informed consent, misconduct, data fabrication and/or falsification, double publication and/or submission, redundancy, etc.) have been completely observed by the authors.

## References

[1] Akhtari-Zavare M Juni MH Said SM (2016). Result of randomized control trial to increase breast health awareness among young females in Malaysia. BMC Public Health, 16:738. 27502284 10.1186/s12889-016-3414-1 PMC4977616

[2] World Health Organization Cancer. http://www.who.int/news-room/fact-sheets/detail/cancer (Assessed 6 Jun 2018).

[3] National Cancer Registry Malaysian national cancer registry report 2007–2011. Ministry of Health Malaysia; 2016 http://nci.moh.gov.my/index.php/ms/pengumuman/340-malaysian-national-cancerregistry-report-2007-2011

[4] Akhtari-Zavare M Latiff LA Juni MH (2015). Knowledge of Female Undergraduate Students on Breast Cancer and Breast Self-examination in Klang Valley, Malaysia. Asian Pac J Cancer Prev, 16: 6231–6235. 26434821 10.7314/apjcp.2015.16.15.6231

[5] Avery M Williams F (2015). The Importance of Pharmacist Providing Patient Education in Oncology. J Pharm Pract, 28(1): 26–30. 25540194 10.1177/0897190014562382

[6] Adamowicz K (2017). Assessment of quality of life in advanced, metastatic prostate cancer: an overview of randomized phase III trials. Qual Life Res, 26: 813–822. 27738867 10.1007/s11136-016-1429-9

[7] Bei Y Li-Ming Y Li-Peng H (2016). Determinants of Quality of Life for Breast Cancer Patients in Shanghai, China. PLoS One, 11(4): e0153714. 27082440 10.1371/journal.pone.0153714 PMC4833339

[8] Quinten C Martinelli F Coens C (2014). A global analysis of multi trial data investigating quality of life and symptoms as prognostic factors for survival in different tumor sites. Cancer, 120: 302–11. 24127333 10.1002/cncr.28382

[9] Movsas B (2003). Quality of life in oncology trials: a clinical guide. Semin Radiat Oncol, 13: 235–47. 12903013 10.1016/S1053-4296(03)00029-8

[10] Montazeri A Milroy R Hole D McEwen J Gillis CR (2001). Quality of life in lung cancer patients: As an important prognostic factor. Lung Cancer, 31: 233–240. 11165402 10.1016/s0169-5002(00)00179-3

[11] Akhtari-Zavare M Mohd-Sidik SH Periasamy U (2018). Determinants of quality of life among Malaysian cancer patients: a cross-sectional study. Health Qual Life Outcomes, 16:163. 30103759 10.1186/s12955-018-0989-5 PMC6090648

[12] Yan B Yang LM Hao LP (2016). Determinants of Quality of Life for Breast Cancer Patients in Shanghai, China. PLoS One, 11(4): e0153714. 27082440 10.1371/journal.pone.0153714 PMC4833339

[13] Kawaguchi T Iwase S Koinuma M (2012). Determinants Affecting Quality of Life: Implications for Pharmacist Counseling for Patients with Breast Cancer in Japan. Biol Pharm Bull, 35: 59–64. 22223338 10.1248/bpb.35.59

[14] Ibrahim NA Ingunn BR Alwan ASA Honore PH (2014). Insights about health-related quality of life in cancer patients indicate demands for better pharmaceutical care. J Oncol Pharm Pract, 20: 270–277. 24081221 10.1177/1078155213505255

[15] Periasamy U Sidik SHM Rampal L (2014). Managing patients on chemotherapy. UPM Press, Serdang.

[16] Periasamy U Sidik SHM Rampal L (2017). Effect of Chemotherapy Counseling By Pharmacists on Quality of Life and Psychological Outcomes of Oncology Patients in Malaysia: A Randomized Control Trial. Health Qual Life Outcomes, 15:104. 28506305 10.1186/s12955-017-0680-2 PMC5433062

[17] Mohd-Sidik SH Akhtari-Zavare M Periasamy U (2018). Effectiveness of chemotherapy counselling on self-esteem and psychological affects among cancer patients in Malaysia: Randomized controlled trial. Patient Educ Couns, 101:862–871. 29336859 10.1016/j.pec.2018.01.004

[18] National Cancer Institute (NCI) 2007 Managing Chemotherapy Side Effects; Chemotherapy and You. http://www.cancer.gov/cancertopics/coping/chemotherapy-and-you

[19] Hasanah CI Naing L Rahman ARA (2003). World Health Organization Quality of Life Assessment: Brief Version in Bahasa Malaysia. Med J Malaysia, 58:79–88. 14556329

[20] World Health Organization, 2004Quality of Life-BREF (WHOQOL-BREF) http://www.who.int/substance_abuse/research_tools/whoqolbref/en/. On February 4, 2017.

[21] Rosner B (2006). Fundamentals of Biostatistics. Boston, Duxbury Press.

[22] National Cancer Institute (NCI), 2012. Managing Chemotherapy Side Effects; Constipation. 2012. on January 20, 2017.

[23] Puteh SHEW Saad NM Aljunid SM (2013). Quality of life in Malaysian colorectal cancer patients. Asia Pac Psychiatry, 5 Suppl 1:110–7. 23857846 10.1111/appy.12055

[24] Bottomley A (2002). The cancer patient and quality of life. Oncologist, 7(2):120–5. 11961195 10.1634/theoncologist.7-2-120

[25] Bagheri H Memorial R Alhani F (2007). Evaluation of the effect of group counselling on post myocardial infarction patients: determined by an analysis of quality of life. J Clin Nurs, 16(2): 402–406. 17239076 10.1111/j.1365-2702.2005.01498.x

[26] Mohamed NE Herrera PC Hudson S (2014). Muscle invasive bladder cancer: examining survivor burden and unmet needs. J Urol, 191:48–53. 23911603 10.1016/j.juro.2013.07.062 PMC4286331

[27] Singer S Ziegler C Schwalenberg T (2013). Quality of life in patients with muscle invasive and non-muscle invasive bladder cancer. Support Care Cancer, 21(5):1383–93. 23238655 10.1007/s00520-012-1680-8

[28] Li MY Yang YL Liu L Wang L (2016). Effects of social support, hope and resilience on quality of life among Chinese bladder cancer patients: a cross-sectional study. Health Qual Life Outcomes, 14: 73. 27153944 10.1186/s12955-016-0481-z PMC4859956

[29] Zabernigg A Giesinger JM Pall G (2012). Quality of life across chemotherapy lines in patients with cancers of the pancreas and biliary tract. BMC Cancer, 12: 390. 22950826 10.1186/1471-2407-12-390 PMC3488526

[30] Jacobsen PB Jim HS (2008). Psychosocial interventions for anxiety and depression in adult cancer patients: achievements and challenges. CA Cancer J Clin, 58: 214–30. 18558664 10.3322/CA.2008.0003

[31] Detmar SB Muller MJ Schornagel JH (2002). Health-related quality-of-life assessments and patient-physician communication: A randomized controlled trial. JAMA, 288: 3027–3034. 12479768 10.1001/jama.288.23.3027

[32] Sharma S Shobha RH Subramanian G Irma M (2013). Evaluation of Impact of Counseling on Quality of Life of Chronic Kidney Disease and Hemodialysis Patients. IJOPP, 6: 57–61.

[33] Lopez-Martin C Siles MG Alcaide-Garcia J Felipe VF (2014). Role of clinical pharmacists to prevent drug interactions in cancer outpatients: a single-centre experience. Int J Clin Pharm, 36: 1251–1259. 25326824 10.1007/s11096-014-0029-4

